# International perspective on guidelines and policies for child custody and child maltreatment risk evaluations: A preliminary comparative analysis across selected countries in Europe and North America

**DOI:** 10.3389/fpsyg.2022.900058

**Published:** 2022-09-07

**Authors:** Jelena Zumbach, Sonja P. Brubacher, Frank Davis, Corine de Ruiter, Jane L. Ireland, Kathleen McNamara, Martta October, Michael Saini, Renate Volbert, Taina Laajasalo

**Affiliations:** ^1^Psychologische Hochschule Berlin, Berlin, Germany; ^2^Centre for Investigative Interviewing, Griffith Criminology Institute, Griffith University, Brisbane, QLD, Australia; ^3^Association of Family and Conciliation Courts California Chapter, Oakland, CA, United States; ^4^Faculty of Psychology and Neuroscience, Maastricht University, Maastricht, Netherlands; ^5^University of Central Lancashire, Preston, United Kingdom; ^6^Private Practitioner, Fort Collins, CO, United States; ^7^Special Services, National Institute for Health and Welfare, Helsinki, Finland; ^8^Factor-Inwentash Faculty of Social Work, University of Toronto, Toronto, ON, Canada; ^9^Charité – Universitätsmedizin Berlin, Corporate Member of Freie Universität Berlin and Humboldt-Universität zu Berlin, Institute of Forensic Psychiatry, Berlin, Germany; ^10^Department of Psychology, University of Helsinki, Helsinki, Finland

**Keywords:** child maltreatment, custody evaluation, risk assessment, evidence-based practice, best practice

## Abstract

Little knowledge exists on how evaluators in child custody and child maltreatment cases are informed by guidelines, the kinds of qualifications required and the types of training provided in different countries. The purpose of this paper is to provide an international preliminary comparison on how child custody and child maltreatment risk assessments are conducted in selected Western countries, and how the assessments are informed by best practice guidelines. Another aim is to increase knowledge on how the guidelines and best-practice standards could be developed further to reflect recent research findings. A total number of 18 guidelines were included in the analyses: four from Canada, five from the United States, three from the United Kingdom, three from the Netherlands, two from Finland, and one from Germany. We conducted a content analysis of the included guidelines in the database, focusing on how the guidelines address the best interest of the child criteria, guidelines for conducting the assessments, considerations for evaluative criteria, and specific guidance for conducting specific assessment procedures (e.g., interviews and observations). Findings show that the qualifications of and training provided to evaluators in child custody and child maltreatment risk evaluations are largely heterogeneous across the countries represented. Guidelines differ in whether and how they highlight the importance of evidence-based practices and scientifically validated assessment measures. Implications are drawn from the review and contextualized by international expert authors in the fields of forensic psychology, and family law. After the content analysis, discussion sessions within the expert group were held. The authors provide both commentaries and suggestions to improve the development of standard methods for conducting both child custody and child maltreatment risk evaluations and to consider a more transparent and judicious use of social science research to guide methods and the recommendations offered within these assessments.

## Introduction

The UN Convention of the Rights of the Child (CRC) came into force on September 2, 1990, more than 30 years ago, and has since been ratified by most countries. The first five of its 41 Articles provide a foundation and an overall framework for children’s rights. Among these, Article 3 states the importance of using the “best interests of the child” standard in all decision-making concerning children. It is a fundamental principle, guiding the implementation of other rights, and it is the most cited Article of the Convention by judicial decision makers [Child Rights International Network (CRIN), 2012]. It is also one of the most complicated and dynamic concepts of the CRC, and its interpretation and application varies depending on how the concept is understood (Vaghri et al., 2022). CRC articles related to child protection, care, and custody are also among the most regularly cited, especially in cases where separation from parents and protection from abuse are considered [Child Rights International Network (CRIN), 2012]. In the case of child maltreatment and neglect, the CRC takes a strict stance (Article 19): states have a duty to take all appropriate measures to protect children from all forms of physical or mental violence, injury or abuse, neglect or negligent treatment, and maltreatment or exploitation. The CRC, and especially the best interest principle, are a background against which to examine the current practice of child custody and child maltreatment risk evaluations.

The Convention is rooted in the general UN principles on the inherent dignity and equal rights of all human beings. In order to accommodate differences, the Convention does not specify definitions of concepts like “physical and mental violence” or the “best interests of the child.” The general comment No. 14 (2013) of the CRC Committee addresses the latter in greater depth, but also emphasizes the flexible and dynamic nature of the “best interest” concept. While some have applauded the Convention on these so-called “open” norms that allows for vagueness in definitions across states, parties, and legal contexts, others have criticized these open norms for leaving ample room for discretion and variability (Rap et al., 2020).

There has been immense growth in scientific research on what children need for their development (e.g., attachment research), especially during the past 30-plus years. There are now also more studies on the ramifications of failing to meet children’s needs (e.g., child maltreatment research) and on how to talk with alleged child victims and promote credible reporting of their experiences (i.e., child forensic interviewing research). Interestingly, in Article 3, the Convention refers to “*standards established by competent authorities.*” Such standards are obviously of particular relevance when they have an impact on legal decisions that involve children, such as parenting arrangements after divorce and child protection measures. Ideally, such standards would be based on scientific evidence, comparable to evidence-based practice in medicine (Sackett and Rosenberg, 1995; Sackett et al., 1996). Yet, concerns have been raised about values and ideologies guiding decisions on children’s best interests, rather than empirical research (Cascardi et al., 2015). While scholars have long argued for the careful analysis of data from empirical research to effectively address children’s best interests, the shift in child advocacy toward balancing values and empiricism has been slow (Cascardi et al., 2015). Standards and guidelines that combine a children’s rights perspective with solid empirical findings may act as tools for professionals and decision makers to better serve children’s best interests in situations where child safety and well-being are at stake.

Child custody and child maltreatment risk evaluation practice continue to evolve since the past 35 years (Benbenishty et al., 2015; Ackerman et al., 2021; Murray et al., 2021), but there remains diversity in how and by whom these assessments are conducted in different countries. In both child custody evaluations and child maltreatment risk evaluations children’s ‘best interests are the foundation for conducting the reports and making recommendations to the courts. Child custody issues involve parents separating, and the cases typically require custody evaluators to consider the best placement and visitation arrangements for the children. In contrast, child maltreatment evaluations require a determination of whether a child has been maltreated, by whom, how to protect the child, and what appropriate interventions will lower the future risk to the child. It is not uncommon for custody issues and child maltreatment issues to overlap (e.g., Saini et al., 2020).

In a number of (predominantly European) countries, the conduct of child custody/visitation evaluations and child maltreatment evaluation is not as separated in practice as is the case in other (predominantly Northern American) countries. Partially due to differences in the legal systems and the structure of courts, child custody and child maltreatment cases can either fall within the responsibility of the same family court or handled by separate court structures (*cf.*, Zumbach and Volbert, 2021). While it is important to consider the unique differences of custody and maltreatment evaluations, it is necessary to operationalize best interest from each perspective, to understand these differences for training purposes, and to ensure these differences are reflected in the guidelines provided to professionals for the conduct of child custody and child maltreatment risk evaluations.

Calls for evidence-informed practice transcend professional and disciplinary boundaries (Horwitz et al., 2014; Herbert and Bromfield, 2019). In some countries, problems around the availability of experts to conduct such evaluations, as well as problems around access to training for new practitioners have occupied the debate (Family Divison Working Group on Medical Experts in the Family Courts, 2020; Craig, 2021). Further, differences in practices are also evident in the overlap of cases in child custody, child maltreatment, and criminal law (e.g., in cases where a family member is accused of violence against a child and child custody proceedings are ongoing).

Based on these observations, we conducted an international review project to provide a systematic international comparison. The aim was to describe how evaluators in child custody and child maltreatment cases are informed by guidelines, the kinds of qualifications required, and the types of training provided in different countries. Another aim was to increase knowledge on how best-practice standards could be refined to reflect recent research findings, thus contributing to the realization of the rights of children. This project is an international collaboration representing various countries with a major publication presence in family law or child abuse risk assessment research and who responded affirmatively to the invitation to participate (Canada, Finland, Germany, the Netherlands, the United Kingdom, and the United States).

First, we aimed to provide country-by-country vignettes on required qualifications as well as the training provided for child custody/child maltreatment evaluators in the individual countries. Second, we aimed to conduct a comparative systematic content analysis of well-established guidelines for the conduct of child custody and child maltreatment risk evaluations in the different countries, in order to compare practice standards. Analyzing existing guidelines from Canada, Finland, Germany, Netherlands, the United Kingdom, and the United States, we aimed to answer the following questions:

- Who developed/published the guidelines and how binding are they?
- Which qualifications of evaluators are set by the guidelines?
- Which types of cases are covered by the guidelines?
- (How) Is the child‘s best interest principle defined?
- Which general recommendations for the assessment are given (e.g., multimethod approach, multidisciplinary approach, etc.)?
- Are specific recommendations given for the conduct of certain steps of an evidence-based assessment (e.g., regarding the child or parent interviewing, behavioral observation, structured risk assessment tools, etc.)?

Third, we held consultation groups with the expert authors based on our findings, focusing on overarching recommendations from an international perspective as well as needs for future research and practice. Our overall aim was to further promote evidence-informed practices in child custody and child maltreatment risk evaluations by sharing the perspectives and experiences of these international experts.

## Materials and methods

### Formation of the research team

JZ and TL initiated the project and reached out to experienced practitioners and researchers, holding key functions in training and professional organizations, and/or showing relevant publications in the field. We contacted 13 potential participants and asked them to additionally refer us to colleagues who might be a good fit for this project. This resulted in a final research team with participants from Canada, Australia, Finland, Germany, the Netherlands, the United Kingdom, and the United States.

### Data collection: Expert information

The experts first provided information for their particular country or jurisdiction on: (a) which professionals conduct expert evaluations in child custody and child maltreatment cases and which types of cases are typically addressed; (b) which training options/requirements are provided for evaluators; (c) existing legal statutes that determine necessary qualifications of evaluators; and (d) existing means to implement an evidence-informed approach in the evaluation process. This information is described for each country in a Supplementary Table S1.

### Data extraction: Systematic content analysis of existing guidelines

In a second step, the experts provided guidelines^1^ for the conduct of child custody and child maltreatment risk evaluations published by professional associations or public institutions in their respective countries with a somewhat binding character. In order to identify the guidelines that have received professional acceptance within the individual countries and that are currently used in practice, professional experience and knowledge about current practice standards in the specific country was indispensable. As guidelines are published in various locations (e.g., scientific journals, websites of professional associations, websites of Ministries, public institutions, etc.), a systematic literature review in scientific databases, following the typical methodology for the conduct of systematic reviews of scientific studies, was therefore not appropriate to answer our research questions.

In addition to the analysis of guidelines, we aimed to provide comparative contextual information on required qualifications, training for evaluators, and quality assurance means for the included countries. The availability of this information in the guidelines is limited and required additional information, which was provided by the experts. To ensure that our analysis was conducted from within this context, we decided to exclude guidelines from countries that were not represented by an expert in the research team. Therefore, this study does not claim to be a systematic literature review of scientific studies, but rather a systematic comparison of guidelines and policy practices from different countries using the background of professional expert information.

In order to be included in the content analysis, guidelines had to be no older than 15 years and had to be targeted specifically at child custody and/or child maltreatment risk evaluations. Thus, we excluded guidelines focusing on forensic psychological practice in a broad sense, such as the American “Specialty Guidelines for Forensic Psychology” (American Psychological Association, 2013a).

We further excluded research reports or systematic reviews of the scientific literature that did not identify clear and precise practical implications for professionals conducting child custody and/or child maltreatment risk evaluations. A borderline case was the United Kingdom research report on assessing parental capacity to change when children are at risk of requiring out of home care (Ward et al., 2014). We decided to include this research report, as it is stated in the text that it is meant to be a reference source for practitioners.

We further excluded handbooks, articles, or other book publications that include suggestions for the conduct of child custody and/or child maltreatment risk evaluations in practice not published by professional associations or public institutions, because they do not reflect a consensus in the professional community.

Published handbooks, which aimed to summarize the results of scientific studies from the field, with no specified formal usage as guidelines, were likewise excluded. The suggestions for practice found in various handbooks were generally in line with the official guidelines but differed widely in whether they provided clear direction for conducting these evaluations.

Guidelines from Finland, Germany, and the Netherlands were provided in their original languages. In order to include those guidelines in the analysis, they were translated into English language by the research team.

We conducted systematic content analysis on the database, choosing the following parameters deemed to be the central components based on literature as well as on consensus among the expert group: Definition of the best interest of the child criterion, general recommendations for the assessment, recommendations for evaluative criteria, and specific recommendations for conducting individual steps in the assessment procedure.

To quantify our results, for each of the parameters, we defined a specific subset of factors that were rated as present/not present for every guideline in a multi-rater approach (see Tables 1–3). In a first step, all included guidelines were independently rated by JZ and TL, and discrepancies were discussed until consensus was reached. In a second step, the other members of the research group double-checked the ratings on the guidelines for their individual countries. Remaining discrepancies were again discussed until consensus was reached. For an in-depth understanding, specific instructions for conducting individual steps in the assessment procedure were extracted from the guidelines and are presented in Tables 4, 5.

**Table 1:** Guidelines for evaluation in child custody/visitation cases.

	**CA**				**USA**			**FIN**
	Canada Court of Queen’s Bench of Alberta, 2019	Newfoundland and Labrador Association of Social Workers (NLCSW), 2007	Saskatchewan College of Psychologists (SKCP), 2019	Ontario College of Social Workers and Social Service Workers (OCSWSSW), 2009	American Academy of Matrimonial Lawyers (AAML), 2013	Association of Family and Conciliation Courts (AFCC), 2006	American Psychological Association (APA), 2010	Scientific Advisory Board of the Finnish Psychology Association, 2018
*General recommendations for assessment*								
Definition of child’s best interest		x		x	x		x	
Apply multimethod approach	x	x	x	x	x	x	x	x
Apply a multidisciplinary approach								x
Include research findings/only scientifically validated measures					x	x	x	x
Include alternative plausible hypotheses					x	x		x
Include supervision		x			x	x		x
*Recommendations for evaluative criteria*								
Assess child’s needs/concerns	x			x	x	x	x	x
Assess parent’s skills/capacity/cooperation				x	x	x	x	x
Assess family conflict/each parent’s perspective		x		x				x
Assess family relationships/interaction		x		x	x	x		x
Assess risk factors								
Assess resources/protective factors	x	x						
Assess child’s development/educational needs/impairments/coping	x			x	x	x	x	x
Assess views of the child		x		x	x	x		x
Assess mental health of children/parents	x			x				x
Assess parent’s personal history/parenting history				x				
Assess availability and use of effective treatment							x	
*Recommendations for assessment steps*								
Interviews with parents	x	x	x	x	x	x	x	x
Interviews with children		x	x	x, w.i.	x	x, w.i.	x	x
Behavioral observation	x	x	x	x	x	x, w.i.	x	
Third party information	x	x		x	x	x	x	
Testing (optional)	x		x	(x), w.i.	x, w.i.	(x), w.i.	x	
Home visits (optional)			(x)	(x)				x

Ct. of Alberta, Canada Court of Queen’s Bench of Alberta; NLCSW, Newfoundland & Labrador Association of Social Workers; SKCP, Saskatchewan College of Psychologists; OCSWSSW, Ontario College of Social Workers and Social Service Workers; AAML, American Academy of Matrimonial Lawyers; AFCC, Association of Family and Conciliation Courts; APA, American Psychological Association; Sci. Advisory Board, Scientific Advisory Board of the Finnish Psychological Association; and w.i., with instruction. For the specific instructions in original wording see Table 4.

**Table 2:** Guidelines for risk evaluation in child maltreatment cases.

	NL	FIN	USA		UK	
	Association of Dutch Municipalities (VNG), 2014	Finnish Police Government, 2019	American Professional Society on the Abuse of Children (APSAC), 2019	American Psychological Association (APA), 2013b	National Institute for Health and Care Excellence (NICE), 2017	Ward et al. 2014
*General recommendations for assessment*						
Definition of best interest (or any citation or reference)	(x)	(x)				x
Apply multimethod approach	x		x	x	x	x
Apply a multidisciplinary approach	x	x				
Include research findings/only scientifically validated measures	x		x	x		x
Include alternative plausible hypotheses/disproving evidence (not explicitly stated, but indicated)		x	x	x		(x)
Include supervision		x		x		
*Recommendations for evaluative criteria*						
Assess history, nature, scope or severity of violence and maltreatment	x	x	x	x	x	
Assess child’s needs/safety	x	x	x	x	x	x
Assess harm of the child/evidence of trauma	x	x	x	x	x	x
Assess parents’ skills/capacity			x	x	x	x
Assess family and environmental factors			x	x	x	x
Assess caregiver-child relationship			x	x	x	
Assess risk factors that are specifically named/defined in the guideline	x		x	x	x	
Assess protective factors/resources/strengths (factors are not specifically named/defined in the guideline)	(x)		(x)		(x)	(x)
Assess interventive measures and support	x			x	x	x
Explore views of the child/include them in decision making	x	x			x	
*Recommendations for assessment steps*						
Interviews with parents	x		x	x	x	
Interviews with children	x	x	x	x	x, w.i.	
Behavioral observation			x	x, w.i.	x	
Third party information	x		x	x, w.i.		
Testing (optional)	x		(x)	x		
*Recommendations regarding specific instruments*						
Use of Risk Assessment Instruments (or reference to, but note limited evidence)	x		x		(x)	(x)
Use of NICHD (or reference to, but note limited evidence)		x			(x)	

VNG, Association of Dutch Municipalities; Police Gov, Finnish Police Government; APSAC, American Professional Society on the Abuse of Children; APA, American Psychological Association; NICE, National Institute for Health and Care Excellence; and w.i., with instruction. For specific instructions in original wording see Table 5

**Table 3:** Guidelines for evaluation in both child custody/visitation and child maltreatment cases.

	NL		GER	UK
	Dutch Ministry of Justice and Security (RvdK), 2021	Dutch Ministry of Justice and Security (FDJ), 2014	Task Force Expert Evaluations in Family Law, 2019	British Psychological Society (BPS), 2016
*General recommendations for assessment*				
Definition of child’s best interest (or any citation or reference)	(x)		(x)	
Apply multimethod approach	x	x	x	x
Apply a multidisciplinary approach (optional)	x		(x)	
Include research findings/only scientifically validated measures		x	x	x
Include alternative plausible hypotheses				
Include supervision (or four eyes principle)	(x)			x
*Recommendations for evaluative criteria*				
Assess child’s needs/concerns/safety	x		x	x
Assess nature, cause, or seriousness of the problems	x		x	
Assess parent’s skills/capacity/cooperation, including violence, substance use			x	x
Assess family conflict/each parent’s perspective			x	x
Assess family relationships/interaction			x	x
Assess risk factors (not specifically named/defined in the guideline)			x	(x)
Assess protective factors/resources (factors are not specifically named/defined in the guideline)			(x)	
Assess family and environmental factors (optional)	x		(x)	x
Assess child’s development/educational needs/impairments/coping	x		x	x
Assess views of the child/include them in decision making	x		x	
Assess mental health of children/parents (optional)			(x)	
Assess personal history/parenting history (optional)			(x)	
Assess availability and use of effective treatment				
*Recommendations for assessment steps*				
Interviews with parents/	x		x	x
Interviews with children	x		x	x
Behavioral observation			x	x
Third party information	x		x	x
Testing (optional)			x	x
Home visits (optional)			(x)	

RvdK, Dutch Child Protection Board; FDJ, Dutch National Framework for Forensic Diagnostics for Youth; Task Force, German Task Force Expert Evaluations in Family Law; and BPS, British Psychological Society.

**Table 4:** Instructions on specific steps of the assessment as provided in the guidelines for evaluation in child custody/visitation cases.

	**CA**	USA	
	Ontario College of Social Workers and Social Service Workers (OCSWSSW), 2009	American Academy of Matrimonial Lawyers (AAML), 2013	Association of Family and Conciliation Courts (AFCC), 2006
Interviews with parents	-	-	-
Interviews with children	“A custody/access assessment should include interviews with and/or observations of all children who are the focus of the assessment, depending on the age and the child’s receptive and expressive language skills. […] Generally, the child should be interviewed individually and separately from the parents in a private setting (in addition to any interviews of the child that may occur in the presence of a parent). There should be a reasonable balance in terms of who is bringing the child to the interview or in whose home the child is being interviewed. A social worker may also choose to interview siblings together, but it is generally advisable to spend some individual interview and/or observation time with each child. If there is a clear imbalance, the social worker should be prepared to explain the rationale for this. The social worker should inform the child of the limits of confidentiality.”	-	**“**Child custody evaluators shall be trained and skilled in interview strategies with children and shall follow generally recognized procedures when conducting interviews with children. Children who are the focus of custody/access disputes shall be interviewed if they have reasonable receptive and expressive language skills. When structuring interviews, evaluators shall consider a range of hypotheses and base their interview strategies on published research addressing the effects upon children’s responses of various forms of questioning. Evaluators shall have knowledge of and shall consider the factors that have been found to strongly affect children’s capacities as witnesses. Evaluators shall have knowledge of and shall follow generally recognized procedures in establishing the structure and sequence of interviews with children. Evaluators shall commence interviews with children by informing them that what they tell the evaluator is not confidential.”
Behavioral observation	-	-	“Each parent–child combination shall be observed directly by the child custody evaluator, unless there is a risk to the child’s physical or psychological safety. Observations of parents with children shall be conducted in order that the evaluator may view samples of the interactions between and among the children and parents, and may obtain observational data reflecting on parenting skills and on each parent’s ability to respond to the children’s needs. In the course of such observations, evaluators shall be attentive to (1) signs of reciprocal connection and attention; (2) communication skills; (3) methods by which parents maintain control, where doing so is appropriate; (4) parental expectations relating to developmentally appropriate behavior; and, (5) when parents have been asked to bring materials for use during the interactive session, the appropriateness of the materials brought. In formulating their opinions concerning the significance of parent–child interactions, evaluators shall consider religious, cultural, ethnic, and lifestyle factors.”
Third party information	-	-	-
Testing (optional)	“It is important to recognize that the testing is conducted within the context of a comprehensive custody assessment, and consequently, the results must be interpreted with caution and seen as hypotheses to be further tested and integrated with the findings of the [family] member’s assessment. No inferences should be drawn from the [psychological] testing alone as to what would be in the child’s best interests with respect to custody and/or access.”	“Some assessment instruments, data-gathering techniques, and tests that are acceptable in health care settings may not meet the evidentiary demands associated with forensic work. If the validity of an assessment technique has not been established in the forensic context or setting in which it is being used, the custody evaluator should describe the strengths and limitations of any test results and explain the extrapolation of these data to the forensic context. Custody evaluators should utilize the standardized procedures associated with each test.”	**“**Evaluators shall utilize assessment instruments and tests in accordance with the instructions and guidance contained in the manuals that accompany the instruments and tests. When utilizing tests, evaluators shall not make substantial changes in test format, mode of administration, instructions, language, or content, unless extraordinary circumstances require that such changes be made. When such changes have been made, evaluators shall have an affirmative duty to articulate the rationale for having made such changes. Evaluators shall not use instruments for purposes other than those for which they have been previously validated. Evaluators shall be mindful of cultural and language diversity and the impact that these may have on test performance and the resultant data.”
Home visits (optional)	-	-	-

OCSWSSW, Ontario College of Social Workers and Social Service Workers; AAML, American Academy of Matrimonial Lawyers; and AFCC, Association of Family and Conciliation Courts.

**Table 5:** Instructions on specific steps of the assessment as provided in the guidelines for evaluation in child maltreatment cases.

	**US**	**UK**
	American Psychological Association (APA), 2013b	National Institute for Health and Care Excellence (NICE), 2017
Interviews with parents	-	-
Interviews with children	-	“Producing a record of conversations with children and young people about child abuse and neglect, and any subsequent interventions as appropriate to their age, developmental stage and language abilities, and accurately representing their words, as well as checking that they have understood and agree with what is recorded (this could include both of you signing a written record) and record any disagreements. Regarding instructions to conduct the assessment, it is stated that concerns about abuse and neglect should be explored with children in a non-leading way, for example by using open questions.”
Behavioral observation	“In evaluating parental capacity to care for a particular child or assessing the child–parent interaction, psychologists make efforts to observe the child together with the parent in natural settings as well as structured settings. However, in cases where the safety of the child is in jeopardy or where the court has prohibited parental contact with the child, this may not always be possible. Psychologists understand that parent–child observations in safe, structured settings may be of limited predictive value for assessing the safety of parent– child interactions outside of such observations.”	-
Third party information	“Psychologists may also attempt to interview extended family members and other individuals when appropriate (e.g., caretakers, grandparents, clinical and social services providers, and teachers). If information gathered from a third party is used as a basis for conclusions or recommendations, psychologists seek to identify the source of the information, corroborate the information from at least one other source when possible, and, if obtained, document the corroboration in the report. If the information cannot be corroborated but is nonetheless relied upon to support conclusions or recommendations, the psychologist acknowledges that the information is uncorroborated.”	-
Testing (optional)	-	-

APA, American Psychological Association; NICE, National Institute for Health and Care Excellence.

### Expert discussion

In the last phase of the study, we held discussion sessions within the expert group that were guided by our findings as well as by the following questions:

- From the perspective of this international expert group, what should and what should not be in the guidelines?
- What overarching recommendations for guidelines on child custody and child maltreatment risk evaluations can be formulated from an international perspective?
- What needs for future research and practice can be identified?

## Results

### Expert information: Child custody and child maltreatment risk evaluations in different countries

Qualifications of and training provided for evaluators in child custody and child maltreatment risk evaluations appear to vary greatly across the countries represented by this analysis. Country-by-country descriptions are presented in the electronic supplements (Supplementary Table S1).

### Comparative content analysis: Guidelines for the conduct of child custody and/or child maltreatment risk evaluations

#### Description of the database

A total number of 18 guidelines were included in the analysis, among them four guidelines from Canada, five guidelines from the United States, three guidelines from the United Kingdom, three guidelines from the Netherlands, two guidelines from Finland, and one guideline from Germany. Most of the guidelines include minimal standards concerning the professional qualifications of evaluators (e.g., behavioral scientist, licensed/registered psychologist, and licensed/registered social worker) and state that evaluators should have specialized knowledge and training in a wide range of topics, including mental health, or substance use problems.

Whereas US and Canadian guidelines are either directed at evaluation of child custody/visitation cases [e.g., Newfoundland and Labrador Association of Social Workers (NLCSW), 2007; Ontario College of Social Workers and Social Service Workers (OCSWSSW), 2009; American Psychological Association (APA), 2010 ^2^], or directed at risk evaluation in child abuse/maltreatment cases [e.g., American Psychological Association (APA), 2013b; American Professional Society on the Abuse of Children (APSAC), 2019], several of the European guidelines do not make this distinction. A number of European guidelines address both, evaluation of child custody/visitation cases as well as risk evaluation in child maltreatment cases simultaneously [e.g., guidelines from the Netherlands; Dutch Ministry of Justice and Security, 2014, 2021, Germany; Task Force Expert Evaluations in Family Law, 2019, and the United Kingdom; British Psychological Society (BPS), 2016]. Of all the guidelines we included in our analysis, eight guidelines are directed at evaluation in child custody/visitation cases, six guidelines are directed at risk evaluation in child maltreatment cases, and four guidelines are directed at both evaluations of child custody and child maltreatment cases simultaneously. Accordingly, results based on the content analysis of the guidelines are described and displayed in three separate sections below.

### Guidelines for evaluation in child custody/visitation cases

Results of the ratings based on the guidelines for evaluation in child custody/visitation cases are displayed in Table 1.

#### Best interest principle

Deciding the best interests of the child is often stated as the primary goal of child custody evaluations, but this concept is rarely specifically defined. Two Canadian guidelines [Newfoundland and Labrador Association of Social Workers (NLCSW), 2007; Ontario College of Social Workers and Social Service Workers (OCSWSSW), 2009] explicitly state that the best interests may mean different things to different people. Some guidelines define the principle by citing other references, such as the relevant legislation and definition of the Children’s’ Law Reform Act [Ontario College of Social Workers and Social Service Workers (OCSWSSW), 2009]. The American Psychological Association (APA)‘s “Guidelines for child custody evaluations in family law proceedings” provide further specification of the best interests of the child from a psychological perspective, stating that “psychologists should weigh and incorporate overlapping factors, such as family dynamics and interactions; cultural and environmental variables; relevant challenges and aptitudes for all examined parties; and the child’s educational, physical, and psychological needs. Issues that are central to the court’s ultimate decision-making obligations include parenting attributes, the child’s psychological needs, and the resulting fit” (American Psychological Association (APA), 2010; p. 864).

#### General recommendations for the assessment

General instructions for the assessment regarding, for example, a multimethod or a multi-informant approach to assessment, are part of all guidelines. Several guidelines emphasize that the choice of the specific assessment tools in the individual case is within the evaluator’s judgment [e.g., Newfoundland and Labrador Association of Social Workers (NLCSW), 2007; Ontario College of Social Workers and Social Service Workers (OCSWSSW), 2009]. Several guidelines specifically suggest working under continuous supervision [Association of Family and Conciliation Courts (AFCC), 2006; Newfoundland and Labrador Association of Social Workers (NLCSW), 2007; American Academy of Matrimonial Lawyers (AAML), 2013].

The most detailed information on the required qualification of evaluators is found in the “Child Custody Evaluation Standards” published by the American Academy of Matrimonial Lawyers (American Academy of Matrimonial Lawyers (AAML), 2013), stating that “custody evaluators should have at least a master’s degree in a mental health field that includes formal education in the legal, social, familial, and cultural issues involved in custody and parenting time decisions, or a juris doctor degree. Custody evaluators who have fewer than 3 years of experience in conducting custody evaluations and have conducted fewer than 20 custody evaluations should seek ongoing supervision from an experienced custody evaluator prior to offering to perform or accepting appointments to conduct custody evaluations” (p. 262).

The American Academy of Matrimonial Lawyers (AAML) (2013) preamble also state that whereas general educational and experience requirement might be fulfilled in large urban areas where mental health professionals are plentiful, “in the rest of the country, where mental health professionals are scarce and economic resources limited, these evaluations may sometimes be conducted by professionals (which may include attorneys) without training in custody evaluations and court appointed lay persons functioning as Guardians *ad Litem* and under the mantle of various Alternative Dispute Resolution methodologies” (p. 257).

The guidelines include limited information on requirements to guarantee an evidence-informed approach to assessment, for example, there are few references to scientific evidence or reliability and validity issues. The guidelines of American Academy of Matrimonial Lawyers (AAML) (2013) provide an exception, specifically highlighting that “in the selection of methods and procedures, custody evaluators should be aware that the use of greater numbers of instruments (particularly when some of those instruments may be of questionable reliability or validity) does not necessarily produce more reliability and validity in the data set” (p. 271). It is further stated that assessment instruments acceptable in health care settings may not meet the evidentiary demands for forensic contexts or their validity might be uncertain. In this case, the custody evaluator should explain the extrapolation of the findings to the forensic context [American Academy of Matrimonial Lawyers (AAML), 2013]. The guidelines of Association of Family and Conciliation Courts (AFCC) (2006) include a broader statement that evaluators shall be mindful of cultural and language diversity and the impact that these may have on test performance and the resultant data.

#### Specific recommendations for evaluative criteria

All the guidelines that are directed at evaluation in child custody/visitation cases name specific evaluative criteria that should underlie any assessment. Evaluative criteria described in guidelines that were developed for evaluation in child custody and visitation cases are targeted around the child’s needs, parenting skills, family conflict, family relationships, the child’s development and coping, as well as the child’s views and preferences.

#### Specific recommendations for the conduct of the evaluation

Almost all guidelines directed at evaluation in child custody/visitation cases mention interviews with parents and children, behavioral observation, and consultations with collateral sources as essential steps when conducting an evaluation. Some guidelines emphasize that the amount of time spent with both parents should be equal or that dual roles (e.g., evaluator/therapist) should be avoided [e.g., Association of Family and Conciliation Courts (AFCC), 2006; American Psychological Association (APA), 2010].

The majority of the guidelines, however, do not specify how to conduct the individual steps in the evaluation. Regarding child or parent interviewing, only some guidelines provide specific instructions on the interview setting (e.g., to interview the child alone or brought by each parent in a custody case, to interview a child only after informing the child of the limits of confidentiality, and to potentially interview siblings together), but to also spend some time for individual interviews [Ontario College of Social Workers and Social Service Workers (OCSWSSW), 2009]. Some guidelines provide further information on what to interview the children or caregivers about, for example, to interview children about relationships with both parents, about the impact of exposure to conflict, or about their views and preferences.

The number of guidelines that comment on how to conduct behavioral observations of parents and children (e.g., structured vs. unstructured observation, use of empirically validated observational coding systems to analyze observed interaction) is even lower. The guidelines of Association of Family and Conciliation Courts (AFCC) (2006) provide an example of rather broad instructions on the conduct of behavioral observation, by mentioning that observational data should reflect on “parenting skills and on each parent’s ability to respond to the children’s needs” (p. 21). The guidelines of Association of Family and Conciliation Courts (AFCC) (2006) are the only ones that present a set of criteria to guide and interpret the observations: signs of reciprocal connection and attention; communication skills; methods by which parents maintain control, where doing so is appropriate; parental expectations relating to developmentally appropriate behavior; and, when parents have been asked to bring materials for use during the interactive session, the appropriateness of the materials brought. Those guidelines further state that in formulating their “opinions concerning the significance of parent–child interactions, evaluators shall consider religious, cultural, ethnic, and lifestyle factors” [Association of Family and Conciliation Courts (AFCC), 2006; p. 21].

The use of psychological tests is not required and mostly recommended as optional. No specific tests are mentioned in any of the guidelines. The guidelines of Association of Family and Conciliation Courts (AFCC) (2006) provide guidance on the use of tests: “evaluators shall utilize assessment instruments and tests in accordance with the instructions and guidance contained in the manuals that accompany the instruments and tests. When utilizing tests, evaluators shall not make substantial changes in test format, mode of administration, instructions, language, or content, unless extraordinary circumstances require that such changes be made. When such changes have been made, evaluators shall have an affirmative duty to articulate the rationale for having made such changes. Evaluators shall not use instruments for purposes other than those for which they have been previously validated. Evaluators shall be mindful of cultural and language diversity and the impact that these may have on test performance and the resultant data” (p. 18). Specific instructions for the assessment that are included in the guidelines directed at evaluation in child custody/visitation cases are displayed in Table 4.

### Guidelines for child maltreatment risk evaluation

Results of the ratings based on the guidelines for child maltreatment risk evaluation are displayed in Table 2.

#### Best interest principle

Similar to the guidelines for evaluation in child custody/visitation cases, the best interest principle is rarely defined any further in the guidelines for child maltreatment risk evaluation. Some specifications of the construct from a psychological perspective are found in the UK research report (Ward et al., 2014). It is outlined that “assessments, analyses, and judgments about the impact of abuse and neglect on children’s development; parents’ understanding of the need to make changes; and their readiness, motivation, and ability to change will be crucial components of decisions about whether it is in the best interest of the child to remain with parents, or to be placed away from home on a temporary or permanent basis” (p. 49).

#### General recommendations for the assessment

General instructions regarding a multimethod or a multi-informant approach to assessment are also part of all the guidelines for child maltreatment risk assessment. Two out of six guidelines specifically suggest working under continuous supervision (American Psychological Association (APA), 2013b; Scientific Advisory Board of the Finnish Psychology Association, 2018; Finnish Police Government, 2019). A multidisciplinary approach to evaluation is also mentioned in two of the guidelines [Association of Dutch Municipalities (VNG), 2014; Finnish Police Government, 2019].

Some guidelines emphasize further that psychodiagnostic procedures must specifically contribute to answer the psycholegal questions, whereas answering the ultimate legal issue is subject to the court’s authority. According to the American Psychological Association (APA) (2013b), “the profession has not reached consensus about whether psychologists should offer opinions regarding the “ultimate issues” before the court—for example, whether psychologists should offer opinions about child placement, termination of parental rights, or the best interests of the child” (p. 28). The American Psychological Association (APA) (2013b) advises psychologists to be aware of the arguments on both sides of this issue and to be able to explain the logic of their position concerning their own practice.

#### Specific recommendations for evaluative criteria

Evaluative criteria extracted from the guidelines for child maltreatment risk assessment focus on assessing the history, nature, and severity of previous maltreatment, the child’s needs and safety, parenting skills, the parent–child relationship, interventive measures and support, as well as risk and protective factors for future maltreatment. Whereas most of these guidelines provide some instruction as to which risk factors should be assessed (e.g., substance abuse or dependency, domestic violence, health status of family members, and the entire family context; American Psychological Association (APA), 2013b), no guidelines provide further instructions on how to assess protective factors. Indeed, less than half of the guidelines mention assessment of protective factors or strengths as part of the evaluation.

The American Psychological Association (APA) (2013b) guidelines are unique by highlighting that not only risks from any reasonably anticipated parental maltreatment needs to be determined, but also the risk deriving from “multiple substitute care placements; maltreatment while in substitute care; inadequate supports or interventions from poorly resourced child welfare systems; prolonged separation from parents, kin, or other primary caregivers who may be adequate caregivers; unwarranted or poor quality institutional care; or other inadvertent but potentially negative consequences of state intervention” (p. 22).

#### Specific recommendations for the conduct of the evaluation

Almost all guidelines directed at child maltreatment risk assessment mention interviews with parents and children, behavioral observation, and consultations with collateral sources as essential steps when conducting an evaluation. The majority of the guidelines, however, do not specify how to conduct the individual steps in the evaluation.

Only one of the six guidelines provides further instructions on the conduct of behavioral observation (American Professional Society on the Abuse of Children (APSAC), 2019). Yet, the instructions are rather general: “When feasible, the professional should observe the child-caregiver relationship. Repeated observations may be necessary to obtain a representative sample of behavior and to recognize patterns of child–caregiver interaction and should be conducted by someone familiar with the developmental stages of children. Some parents may not behave in their usual manner when being observed, although this is less of a concern the longer the duration of the observation or greater the frequency of repeated observations. The challenge of discriminating between poor or inadequate caregiving and psychological maltreating caregiving can be challenging” (American Professional Society on the Abuse of Children (APSAC), 2019; p. 9).

Few guidelines reference research-based interview protocols, such as the National Institute of Child Health and Human Development (NICHD) interview protocol [National Institute for Health and Care Excellence (NICE), 2017; Finnish Police Government, 2019]. The UK guidelines of National Institute for Health and Care Excellence (NICE) (2017) stand out by stating that for communicating with children and young people, a range of methods should be used, such as drawing, books, or activities, if appropriate.^3^ Specific instructions that are given on the individual steps of the assessment in child maltreatment risk evaluations are displayed in Table 5.

The use of risk assessment instruments that follow an actuarial or structured professional judgment approach is commented upon in four out of six guidelines. The APSAC guidelines include an unvalidated worksheet for structured collection and interpretation of information. According to the Dutch VNG guidelines [Association of Dutch Municipalities (VNG), 2014], the information gathered should be analyzed and always assessed by means of risk assessment using an (evidence- or practice-based) risk assessment instrument. It is not specified further which specific tools should be applied. Two of the guidelines emphasize that the available evidence on the reliability and validity of risk assessments instruments in a child maltreatment risk context is limited [Ward et al., 2014; National Institute for Health and Care Excellence (NICE), 2017]. The NICE guidelines assessed the evidence for a screening checklist that was developed for emergency departments based on a systematic literature review, earlier tools, and interviews, and found the evidence to be poor. Further, Ward et al. (2014) cited Barlow et al. (2012)who in their systematic research found tentative support for the use of one actuarial risk assessment tool in some contexts: the California Family Risk Assessment Tool. Yet, Ward et al. (2014, p. 53) conclude: “A range of materials have been developed to assist practitioners in assessing the likelihood of current or future significant harm. Most of these need further validation and/or translation and piloting in a UK context. Once properly validated they should provide practitioners with standardised measures that can support the development of structured professional judgment.”

### Guidelines for evaluation in both child custody/visitation and child maltreatment cases

Results of the ratings based on the guidelines for evaluation in both child custody/visitation and child maltreatment cases are displayed in Table 3.

#### General recommendations for the assessment

The instruction to apply a multimethod approach to assessment is part of all the guidelines for evaluation in both child custody/visitation and child maltreatment cases. In the Dutch guidelines of the Child Protection Board (RvdK), it is explicitly stated that their working method is multidisciplinary. The guideline also takes a position regarding the roles of different disciplines and professionals in the multidisciplinary collaboration: behavioral science expertise should be used in all cases of serious and complex developmental and child-rearing problems, and a qualified behavioral scientist should be involved in decisions about out-of-home placement (Dutch Ministry of Justice and Security, 2021). However, the exact qualifications in relation to the required level of knowledge and skills (for instance, concerning child investigative interviewing, parent–child observations) are not specified.

A multidisciplinary approach to evaluation is mentioned as optional by the German Task Force Expert Evaluations in Family Law (2019). The guidelines of the German Task Force Expert Evaluations in Family Law (2019) further highlight that the court is responsible for subsuming the evaluator’s recommendations under legal categories and constructs.

#### Specific recommendations for evaluative criteria

Evaluative criteria listed in general evaluation guidelines for both child custody as well as child maltreatment cases focus on assessing the child’s needs, safety, and development, family conflict, parenting skills, family relationships, the views of the child, and risk factors and resources rather broadly. These general guidelines do not specify how different evaluative criteria need to be weighed in relation to different case constellations [Dutch Ministry of Justice and Security, 2014, 2021; British Psychological Society (BPS), 2016; Task Force Expert Evaluations in Family Law, 2019].

#### Specific recommendations for the conduct of the evaluation

The general guidelines provide varied information on the essential steps when conducting an evaluation. The Dutch Child Protection Board (RdvK; Dutch Ministry of Justice and Security, 2021) mentions interviews with parents and children as well as third party information as essential sources of information, and the Dutch National Framework for Forensic Diagnostics for Youth (Dutch Ministry of Justice and Security, 2014) provides no further information on the conduct of the assessment. The German Task Force Expert Evaluations in Family Law (2019) and the British Psychological Society (BPS) (2016) agree on stating that interviews with parents and children, behavioral observation, and collecting third party information are essential, and psychological testing as well as home visits are optional steps when conducting an evaluation. None of the general guidelines provide specific instructions on how to conduct the individual steps in the evaluation. These findings echo research conducted elsewhere, calling for more concrete and specific guidelines around how the different parts of these evaluations should be conducted (e.g., Australia; Turoy-Smith and Powell, 2017; Turoy-Smith et al., 2018).

### General findings across all guidelines

Only 11 out of the 18 guidelines state that evaluators should include empirically validated research findings or use only scientifically validated measures when conducting their evaluations. Even fewer guidelines (10 out of 18) comment on the necessity to investigate alternative hypotheses or scenarios, or to apply the principle of falsification rather than verification when conducting the assessment.

#### Avoiding biases and stereotypes

The effects of the evaluators’ biases, attitudes and preconceptions emerged as one of the key themes to be addressed. The most comprehensive coverage of the topic was found in the North American guidelines. For example, in the AAML (2010) guidelines, recognizing and preventing the effects of bias, attitudes, values, beliefs, and opinions are mentioned multiple times, and this is seen as a prerequisite for a balanced outcome. Both guidelines for psychologists of American Psychological Association (2010, 2013b) include directions regarding the consideration of the impact of personal beliefs, experiences, and societal prejudices. Psychologists appreciate that preconceptions and biases may significantly impact their work, particularly in circumstances when they may prematurely believe a particular conclusion is obvious—an example of confirmatory bias. The guidelines of Association of Family and Conciliation Courts (AFCC) (2006) stated that knowledge of sources of evaluator bias should be one of the educational requirements. Superficial coverage was found in some of the other countries’ guidelines (United Kingdom, FIN, and NL). For example, UK BPS guidelines (2016) mention triangulation of data as a method to overcome bias of individual methods.

## Discussion

This study is the first to provide a systematic international comparison of best-practice guidelines for conducting child custody and child maltreatment risk evaluations. This research was conducted in a collaboration of researchers and practitioners from Australia, Canada, Finland, Germany, the Netherlands, the United Kingdom, and the United States.

Qualifications of, and training provided to, evaluators in child custody and child maltreatment risk evaluations appear to vary largely across the countries represented by this group of experts. Thus, even though this review did not include all possible countries, the evidence points to heterogeneity of these evaluations around the world. Systematic content analysis of existing guidelines on the conduct of child custody and child maltreatment risk evaluations showed similarities, as well as differences. Generic instructions for the assessment are part of all the guidelines (e.g., using a multimethod or a multi-informant approach to assessment). However, only 11 out of the 18 guidelines we analyzed specifically state that experts should include empirically validated research findings or use only scientifically validated measures. Whereas one could argue that this should be evident without the need to specifically state this in the guidelines, research has shown that not only scientifically validated measures have been used in practice (e.g., projective techniques; Bow and Quinnell, 2001; Ackerman and Pritzl, 2011; Zumbach and Koglin, 2015; Ackerman et al., 2021; Erens et al., 2022). Only 10 out of the 18 guidelines comment on the necessity to investigate alternative hypotheses or to apply the principle of falsification rather than verification, when conducting the evaluation (Lubit, 2021).

All guidelines identify specific evaluative criteria that should underlie all assessments. However, heterogeneity exists in the specification of evaluative criteria across guidelines. Little attention has specified the application of different evaluative criteria according to special case-based considerations. For example, family law professionals have commented on the wide range in complexity of cases they face on a continuum, from seeing how children are adjusting to a recent parental separation, to making assessments in chronically toxic post separation family environments characterized by violence and vitriol (Turoy-Smith et al., 2018).

Regarding the different steps in a multimethod assessment, almost all guidelines mention interviews with parents and children, behavioral observation, and consultation with collateral informants as essential steps. The majority of the guidelines, however, do not specify how to engage in data collection within the assessment. Only a few guidelines provide specific instructions to conduct child or parent interviews and little attention regarding guidelines for conducting and interpreting findings from behavioral observations. Evidence-based interview protocols, or evidence-based observational coding systems are scarcely referred to. The effects of the evaluators’ bias, attitudes and preconceptions emerged as one of the key themes to be addressed in these evaluations, but only few of the guidelines provide guidance on controlling these potential biases.

### Expert group discussion

Based on our findings, we held a discussion with the expert group, focusing on three questions: (1) From the perspective of this international expert group, what should and what should not be in the guidelines? (2) What overarching recommendations for guidelines on child custody and child maltreatment risk evaluations can be formulated from an international perspective? (3) What needs for future research and practice can be identified? The results of this discussion are summarized in the sections below.

#### Question 1: From the perspective of this international expert group, what should and what should not be in the guidelines?

A key question we identified was whether the guidelines should include additional instructions or “*How-To’s”* (i.e., specific instructions on the individual steps of the child custody or child maltreatment risk assessment). Research can provide us with evidence on how to conduct certain steps of the assessment in the given context, such as child and parent interviewing or psychological testing, and guidelines can benefit from a strong link to those research findings when formulating recommendations. On the other hand, guideline developers likely want to avoid an overly-prescriptive approach that becomes inflexible to the needs of the specific case(s). Rather, they should suggest good practices, with useful guidance that could assist the evaluator in ensuring a thorough assessment.

*The problem is that the guidelines may get too long and developing them, you try to balance a lot of considerations. However, the American Task Force that is currently working on the revision of the AFCC guidelines decided to include some basic “How-To’s* “*in the revision, like recommending open ended questioning when conducting child interviews, or understanding the factors that impact child witnesses. Also, we do name, for example, the responsibility to recognize existing interview protocols. But this is pretty general. Maybe we need to be really more thoughtful about that, which “How-To’s “make it into the guidelines and which do not (McNamara, USA).*

*In order to be accepted as useful by the field, the guidelines need to strike a careful balance between relative generality, as to feel applicable to the variety of individual cases, and yet simultaneously contain enough details on evidence-based tools on how to actually conduct the assessment, in order to reach a satisfactory level of uniformity regarding the quality of the assessments (October, Finland).*

Evidence-informed recommendations on conducting certain steps of the assessment can only be given when there is a sufficient body of research, or they become too speculative for application. Guidelines should highlight on how best to integrate behavioral and social science research into the assessment process.

*The important thing is that one realizes in which field evidence-based information is available. Not all fields relevant to family law assessments have been studied extensively. In some fields, such as child interviewing, we can recommend what a best approach might be, because there is a lot of research on child interviewing related to child maltreatment, and less, but also some research on child interviewing in custody related questions. However, for example, the conduct and interpretation of behavioral observations in the given context is a field where we are really lacking this evidence-based information. It would be helpful to systematically identify the fields, in which research-based information is available, and areas in which it is not (Volbert, Germany).*

*Forensic risk assessment is a field that has evolved significantly and guidelines can highlight this, directing assessors to focus more on structured clinical risk assessment tools and incorporating protective assessments, where possible. Highlighting this as evidence-based and accepted practice, including direction to seminal papers, is both useful and important for expert assessments. Equally, guidelines can direct on where there are remaining gaps of knowledge, so that courts are given a critically evaluative view of the field. For example, highlighting how the protective assessment area is continuing to develop and grow empirically, but is not yet equal to the structured risk assessment area (Ireland, UK).*

##### Child investigative interviewing and use of the NICHD protocol

There remains a large gap between the existing and cumulative social science research and the existing guidelines for child investigative interviewing. For child interviewing in maltreatment investigations, there is relatively strong evidence to support techniques, methods, and approaches. On the other hand, for child interviews conducted in the context of custody evaluations, there is limited empirical evidence.

Several structured interview protocols and guidelines have been developed for children alleging maltreatment, based on several decades of research on how to best question children about their experiences. A non-exhaustive list of example interview protocols includes the National Institute of Child Health and Human Development (NICHD) protocol (Lamb et al., 2018); Tom Lyon’s 10 step version (Lyon, 2005); the Standard Interview Method (SIM; Powell and Brubacher, 2020); the Forensic Interview Structure of National Children's Advocacy Center (2019); Guidance for Achieving Best Evidence (ABE) in Criminal Proceedings (Ministry of Justice, 2011), and local protocols, such as the State of Michigan Governor’s Task Force on Child Abuse and Neglect and Department of Human Services (2017).

The NICHD protocol has the strongest research base specifically on the protocol itself (Olafson, 2012; La Rooy et al., 2015). Most of the other interview guidelines mentioned above are based on the same principles. In essence, these principles involve exhausting children’s accounts with open-ended questions; maintaining a hypothesis-testing, unbiased approach; being non-judgmental; offering non-contingent support; and using simple and developmentally-sensitive language (Powell and Snow, 2007; Newlin et al., 2015; Poole, 2016; Powell and Brubacher, 2020). These best-practice principles are the hallmark of high-quality interviewing, not the specific protocol or framework. This means that guidelines can be adapted to various contexts, such as interviews conducted by custody evaluators for decision-making purposes—as long as interviewers are well-trained and receive regular feedback (see Powell and Brubacher, 2020, for discussion of interview adaptations). The NICHD protocol has been adapted for use in various contexts internationally (La Rooy et al., 2015).

*In the Netherlands, the NICHD protocol is used quite often not only in child maltreatment risk evaluations, but also in cases of a child caught in the middle of a high conflict divorce between their parents. In my opinion, the interview works very well in those cases. Everybody thinks that you need a specific abusive event to ask the child about in order to apply the NICHD protocol. But you can also just ask a child whose parents are divorced questions like “With whom do you live most of the time?,” “How are the transitions going?,” “Tell me about your last transition, when you went from your Mom’s to your Dad’s.” And then you go deeper into the event. By using the NICHD as a semi-structured protocol you get much more information about the child and the actual situation it finds itself in (de Ruiter, Netherlands).*

*I think the application of the NICHD works quite well in crossover cases, where both child maltreatment risk and a custody dispute are at question. I think it is often forgotten that it is really a half-structured protocol. This means you can add things, and of course, if you are looking at custody-related questions, you have to modify it. However, the basic structure is very helpful. In Finland, we are currently developing an interview-model for custody cases. It includes certain elements from the NICHD, such as the practice narrative and a set of conversational rules, but it is less focused on event-specific questions and also includes more generic questions of routines and relationships, for example. The instructions we have included are quite general like “Use time for rapport-building; Be aware of the question types you use.” It is essential to keep in mind the hypothesis testing approach and you can really use the NICHD approach to test alternative hypotheses (Laajasalo, Finland).*

*If you are aware of the research on the NICHD, it is much easier to adapt it to a child custody evaluation. If you are not aware of this research, then you might misunderstand the protocol as a set of questions which is then difficult to adapt to this new situation. It is more the idea, the whole mindset behind this protocol, than the specific line of questioning that makes it so valuable (Volbert, Germany).*

##### Behavioral observations

Observations of parent–child interactions in natural or structured settings are a crucial method when evaluating constructs such as parenting skills, parent–child interaction problems, and attachment quality (Bennett et al., 2006; Harnett, 2007; Zumbach et al., 2021). This especially applies to cases of young children who are limited in their receptive and productive language competencies when investigative child interviews may not be sufficiently informative. It is a common assumption that stable patterns in a relationship are reflected in parent–child interactions. There are a wide variety of formats, procedures, and techniques that evaluators use to assess parent–child interactions and behaviors in the given context (Saini and Polack, 2014).

The literature shows that the systematic nature of a behavioral observation has a large impact on its reliability and validity (Hynan, 2003; Saini and Polack, 2014). Observations of parents and children potentially yield a wealth of information, and it is important for evaluators to consistently apply scientific principles to make sense of that information (Hynan, 2003). This speaks for the application of structured and semi-structured observational coding systems (Haynes and O’Brien, 2000).

A number of observational coding systems have been developed to examine relevant constructs, such as attachment quality, parenting skills, or the interaction between parent or caregiver and the child. Several well validated coding systems exist that were developed to detect child maltreatment risk in parenting behavior, such as the Child–Adult Relationship Experimental Index (CARE-Index, Crittenden, 2004, 2006), and the Psychological Maltreatment Rating Scales (PMRS; Brassard et al., 1993). Other coding systems with satisfactory to high reliability and validity focus on parenting behavior or the parent–child interaction more broadly, such as the Parent/Caregiver Involvement Scale (P/CIS; Farran et al., 1986), the Parent–Child Interaction Procedure (Heller et al., 1998), and the Parent–Child Interaction Coding System-II (DPICS-II; Eyberg et al., 1994).

While there have been considerable efforts to develop structured observational coding systems, not all of these may be appropriate to assess child maltreatment risk or custody-related questions (Budd and Holdsworth, 1996; Budd, 2001; Forslund et al., 2022). The research about the reliability and validity of existing coding systems for the given forensic contexts is very limited. Few studies report on parent–child observations within the context of child custody evaluations (Saini and Polack, 2014).

*We do not have the same evidence-base for behavioral observation in a forensic setting that we have for child interviewing. There is some literature, for sure but not nearly what we have for child interviewing. And many people do not know that literature. There are behavioral coding devices, but they are mostly not specific to child custody. There are some coding devices out there, but hardly anybody uses them, and if they do, applications vary widely. We have to keep in mind that our child custody evaluations are really quite separate from our child maltreatment investigations. Although you cannot completely separate them, because people bring up allegations about poor quality parenting in child custody evaluations, and when suspecting child abuse or neglect, you are obligated to report it. But in both settings, we are observing parents and children all the time. We are expected to do this as a standard procedure, but how professionals do this is very heterogeneous. This really is an area which is underdeveloped (McNamara, USA).*

On the basis of our review of the existing guidelines, the current evidence and the expert discussion, it can be concluded that the choice of an observational measure for a child custody or a child maltreatment risk assessment context is not straightforward. Along with factors influencing the selection that lie within the individual case (such as the age of the child), the relative value of parent–child observations should take into account the discriminant validity, as well as the position and value of parent–child observations within the context of the larger evaluation. Furthermore, using a structured observational coding system requires extensive training and regular feedback. The development of relevant observational measures and their validation are a challenge for future research in order for such measures to be of use in child custody and child maltreatment risk evaluations.

##### Maltreatment risk assessments vs. custody assessments

Child custody and child maltreatment risk evaluations are not mutually exclusive. So-called crossover cases are not uncommon: allegations of child maltreatment may surface within the context of child custody disputes, and these allegations must be carefully assessed by authorities (Saini et al., 2020). In some European countries, such as Germany, the Netherlands, or the United Kingdom, the conduct of both child maltreatment risk and child custody assessments appears to be quite intertwined, whereas in other countries, such as Finland, the US and Canada, these are two separate areas of professional activity. This is also reflected in the guidelines we examined in our analysis, as some German, Dutch, and United Kingdom guidelines are directed at both child custody as well as child maltreatment risk assessment, whereas the US, Canada, and Finland have separate guidelines for the conduct of forensic assessments in each context.

Differences appear to be at least partly due to differences in the legal systems. In Finland, for example, administrative courts handle cases related to child welfare (e.g., out of home placements), whereas custody cases and maltreatment cases are handled in general courts, but separately: the former as civil cases and the latter as criminal cases. Unlike in many other countries, the great majority of child maltreatment allegations, including cases of corporal punishment, are investigated by the police in a pretrial investigation process, where the child is in a position of an injured party, which is governed by specific pretrial investigation legislation (Criminal Investigation Act, 805/2011; for further country-by-country descriptions see Supplementary Table S1). The challenges posed by parallel, but separate family law, criminal law and child protection procedures, as well as the varying definitions of the children’s best interests in the different legal systems have been previously discussed (e.g., Bala and Kehoe, 2013; Hiitola and Hautanen, 2017).

The differences among the legal systems should be taken into account when considering best-practice guidelines to serve the evaluation of cases that intersect different systems. When child maltreatment is assessed in a criminal law context, the legal proceedings, as well as the burden of proof, are different from custody or child welfare cases. In the former, the focus of the proceedings is on defining the legal responsibility of the defendant, and the requirements of a fair trial, “proof beyond a reasonable doubt,” as well as the legal protection of both parties. This has implications for various stages of the evaluation, for example, the way the child’s wishes can be respected. In comparison, in the custody and child welfare proceedings the evaluation is more directly guided by the principle of the child’s best interests. Best-interest decisions are about balancing probabilities and predicting children’s best probable futures (Forslund et al., 2022), whereas maltreatment assessments in the criminal context focus on what happened in the past.

##### Best interests of the child and evaluative criteria

The overarching principle that guides decision making in the context of child custody and also child abuse risk evaluations is the best interests of the child principle. This is a legal principle that derives from the UNCRC. It calls for the determination of a variety of factors that best suit the child’s needs in a specific situation (Miller, 2002), and the purpose is to ensure the full and effective enjoyment of all rights and the holistic development of the child, as defined by the CRC. The principle emphasizes the importance that cases should be decided with the child’s interest foremost and not that of the parents. What is in the child’s best interest, however, often becomes a matter of contention (Ladd, 2017).

The best interest concept, as listed in the guidelines we analyzed, varied considerably. There was no clear consensus defining the dimensions and variables to be evaluated when conducting assessments to determine the child’s best interests, which is also reflected in the literature (Gould, 1999; Gould et al., 2016). Further, similar indeterminacy can be seen, for example, in child protection legislation in various countries. It has been stated that the current emphasis on individual case consideration combined with lack of standards and strong discretion, may lead to (large) discrepancies between professionals who advise in cases of potentially life-altering decisions, such as supervision orders and out-of-home placement (Skivenes and Sørsdal, 2018).

As highlighted by the Commission on European Family Law, the best interest of the child is not a fixed notion, but must reflect the prevailing values in the society in question as well as the specific situation of the child (Boele-Woelki et al., 2007). It is a complex concept, which needs to be determined on a case-by-case basis. The judge, administrative, social, or educational authority will be able to clarify the concept and make concrete use thereof, only if the assessment is conducted on an individual basis, according to the specific situation of the child or children concerned, and taking into consideration their personal situation and needs (Ruggiero, 2022). The ultimate best interest decision remains within the responsibility of the court and not within the responsibility of the psychological evaluator. Yet, it has also been stated the best interest standard is dependent on research, and has infused developmental psychological theories, such as those related to attachment, into the court context (Forslund et al., 2022).

From a psychological perspective, the child’s best interests are usually operationalized by a number of factors related to the child’s circumstances and the caregiver’s circumstances and capacity to parent, with the child’s ultimate safety and well-being being the paramount concern. Commonly named factors in the international literature include the relationship and attachment between the child and caregivers, mental and physical health needs of the child, the child’s need for stability and continuity, the preference and wishes of the child, the parenting capacity, parenting skills, and educational skills as well as the mental and physical health of the parents. Nevertheless, the factors listed in different sources vary considerably (Gould, 1999; Child Welfare Information Gateway, 2016; Gould et al., 2016), which is also reflected by the guidelines we examined.

History of maltreatment not only with the child, but also with other family members such as siblings is addressed in the guidelines implicitly only. The evaluators are instructed to take into account domestic violence, family conflict, and/or the evaluator may be asked to assess and describe sibling relationships. Yet, parent-sibling or sibling-sibling violence/maltreatment is not mentioned *per se*. This stands in contrast to the fact that recent studies also acknowledge exposure to parent assault on a sibling as an often forgotten childhood adversity (e.g., Tucker et al., 2021).

*What struck me when I looked at our results was how few of the guidelines actually define the best interest criterion, or describe evaluative criteria based on psychological best interest definitions. Some guidelines reference the UNCRC or other legal statutes, which makes sense, but in addition to that, a psychological definition of the child’s best interest criterion was mostly lacking. What I was missing in the guidelines was introductory information on what best interest definitions the guidelines are based on, and what evaluative criteria are chosen accordingly that can then be used to structure the suggested assessment and plan the individual steps. Mostly, you can find this somewhere spread around the guidelines, but it often remains unclear as for which criterion which assessment step should contribute information. This is especially important for the general guidelines that are simultaneously directed at both, child custody and child maltreatment evaluations. Those should include a specification on how to include or weigh different psychological best interest criteria for example when assessing what serves the child’s interest better (*e.g.*, in custody related questions)* vs. *assessing a threat of harm to the child (*e.g.*, in child protection matters). (Zumbach, Germany).*

*What we have in the guidelines is a number of assessment methods and then we have some evaluative criteria. However, this is somehow often not very well connected. In some of the guidelines, the assessment methods are even mentioned before the evaluative criteria. This should be described the other way around. We should first determine which information on what criterion is needed and describe second how to collect this information. This should be the basis of any guideline. To some extent it sometimes even remains unclear what we are really expected to predict. Is this the future well-being of the child, or is it future parental behavior? It should be clear what is in the focus of the prediction before recommendations for the assessment are given (Volbert, Germany).*

#### Question 2: Overarching recommendations from an international perspective?

Ultimately, there is a need for balanced guidelines, those that recognize both strengths and weaknesses of any given approach to assessment. This would allow for more balanced assessments and assist with cross-examination and the court’s decision making. According to surveys, methods that do not fulfill the requirements of forensic assessments, such as projective techniques, are still used in custody assessments (Ackerman et al., 2021; but see Viglione et al., 2022 for a different view). Guidelines should take a sufficiently clear position against scientifically outdated or invalid methods in the context of assessments. For example, in one of the reviewed guidelines for this article, drawing and the use of props were mentioned as ways to enhance communications with children [National Institute for Health and Care Excellence (NICE), 2017]. In the context of forensic assessment, however, one should be careful not to rely on drawings, play or other non-direct forms of obtaining information from children to overcome their cognitive and emotional limitations. Further, guidelines should be careful not to promote over-simplified and non-scientific accounts of popular concepts, such as attachment, as has been noted for some social work guidelines (Forslund et al., 2022).

Similar to guidelines regarding the utilization of psychological tests across forensic contexts (see the Special Issue on Personality Assessment in Legal Contexts of *Journal of Personality Assessment*; Neal et al., 2022), the aim should be to establish a set of concrete guiding principles based on relevant literature, which would likely enhance the objectivity and validity of the evaluations related to family law. However, for most assessment questions, “cookbook” guidelines are not feasible (see our discussion regarding Question 1), as the situations are highly complex and non-uniform, and the evaluation process cannot be reduced to binary choices. A recent example of a generic type of best practice recommendation suited for international purposes are the “rudimentary guidelines” presented for assessments of parental alienation in family law (Johnston and Sullivan, 2020). On the other hand, although guidelines cannot provide protocols for all phases of the assessment, they should clearly indicate where scientific evidence provides specific and more solid tools for the evaluator, such as is the case for child investigative interviewing.

#### Question 3: Needs and recommendations for future research as well as for the implementation of the guidelines into practice

We identified several areas related to child custody and child maltreatment risk assessments that, in our opinion, would benefit from increased research efforts that might then subsequently inform future revisions of the guidelines. For example, although best practice principles for child forensic interviewing are largely the same for different types of evaluations, ideally guidelines would include adaptations of current protocols for different case constellations. To achieve this, more research on adapting the existing interview protocols is needed.

Furthermore, there is an urgent need to conduct research regarding objective coding measures to improve the assessment of parent–child observations and ways to limit the threats to the validity of these observed interactions (Saini and Polack, 2014). Associated with this might be the need for increased efforts to study constructs such as parenting behavior indicating child maltreatment risk, or parenting behavior in relation to high conflict separations.

From a broader perspective, future research could consider both the quality of assessments but also their usefulness in terms of the impact they had on decision-making by the court. There is a rightful focus on quality but the utility of the assessments to the legal decision makers also requires empirical investigation. Ultimately, the expert is to assist the court and so far, little is known of the value of expert assessments to decision-making (Waller and Daniel, 2004).

Lastly, studies show that forensic psychologists frequently see introspection and awareness as adequate remedies against evaluator bias, despite evidence showing otherwise (MacLean et al., 2019). More research should be conducted on how to effectively minimize the effects of cognitive biases in child custody and child maltreatment evaluations (Zapf et al., 2018), many of which relate to sensitive issues such as ethnicity, gender or culture (Maldonado, 2017). Some of this information could then be incorporated into the guidelines. Currently, the effect of biases is acknowledged in a minority of the guidelines, but there is very little knowledge on how to mitigate them.

Regarding the implementation of the guidelines into practice, success is largely determined by their acceptance by practitioners. In several of the countries represented in this work, the acceptance of guidelines has been subject to a multidisciplinary debate, also targeting questions on how both national as well as international research evidence can be best made available to practitioners in the field.

*Any translation of guidelines into practice requires an acceptance of their value by those recommending and/or using them. Otherwise, they become nothing more than a paper exercise, unless enforced by a court as part of procedural guidelines (Ireland, UK).*

As guidelines typically include recommendations but no binding requirements, the question remains how to integrate effective evidence-informed guidelines into practice norms. Recommendations can be derived from research, but many times, the training and ongoing feedback that is provided to practitioners is crucial to these recommendations actually being translated into practice (Cyr et al., 2012; Brubacher et al., 2021).

*The thing is, that science teaches us that, for example, just telling people to ask open-ended questions is not effective. People need to be trained and they need to receive feedback on the training interviews. There are a few interview protocols with good evidence and the research shows that they actually do lead to more accurate statements of the children, but only when adequate training and feedback is provided (de Ruiter, the Netherlands).*

*Given the diversity of circumstances that custody evaluators will face, and that guidance materials for assessment are likely to be a set of key steps and principles rather than structured or semi-structured interview protocols, high quality training will be paramount. Training should be congruent with what we know about human learning - the content and learning goals should be clearly articulated, learners should have multiple practice opportunities to apply their skills spaced over time and with different contexts (*e.g.*, different scenarios), they should obtain feedback on their developing skills, and they should learn to objectively evaluate their own performance. These factors can increase the depth of their learning and their retention of skill (Brubacher, Australia).*

The systematic comparative analysis of guidelines for conducting assessments provide important considerations for implementing and/or refining guidelines into practice. This project, however, is not without limitations. We only included perspectives and guidelines from several European countries (Finland, Germany, Netherlands, and United Kingdom) as well as the United States and Canada. Despite our efforts to include contributions from other countries (e.g., Australia, Japan, and Israel), we had to decide to start the project at some point. Clearly, our results are not generalizable beyond the countries included in our analysis. Furthermore, some of the guidelines we included in our analysis are currently undergoing revision (e.g., AFCC and APA guidelines for child custody evaluation). If and how these revisions may incorporate some of the suggestions presented in this paper remains open to further analyses. Also, this work largely focuses on the perspective from mental health professionals. Additional collaboration with legal professionals may further enhance the legal parameters of these assessments. For example, from a legal point of view, the guidelines should acknowledge not only the obligations under the CRC, but also the national law requirements and definitions related to the best interest principle, which in many countries may be fragmented and unclear. Finally, conducting an assessment is only one step in the process. High-quality report writing and a transparent, objective presentation of the results is especially important in cases where the report influences the court’s decision, but a review of this topic was beyond the scope of this article.

### Conclusion

Professional guidelines do not come without limitations. In the field of medicine, well-documented concerns regarding the trustworthiness of the guideline development process have been raised (e.g., Cosgrove et al., 2017). Intellectual conflicts and confirmatory bias may influence guideline developers when adherence to a specific point of view serves academic interests or the aims of an advocacy group (e.g., Cosgrove et al., 2013). Scholar-advocacy bias is a recognized problem in the field of social science research related to family law (Emery et al., 2016). It would be naive to assume that these identified biases will not find their way into professional guidelines for child maltreatment and child custody evaluations if safeguards are not put in place (e.g., by balancing the professional and research backgrounds of the guideline development task force).

Despite the limitations noted, guidelines for conducting assessments do have their place to improve the overall quality, utility, and effectiveness. Surveys among various professionals show that child custody practice has improved over the years, and forensic and child custody guidelines have likely had an effect (Ackerman et al., 2021). Guidelines developed by professional bodies and organizations can bring together the views of its members in the creation of these guidelines, as well as integrate recent and relevance scientific evidence.

Since our results show such heterogeneity across the select Western countries, and research is underdeveloped in many related areas, it would be premature to develop international “checklists” as to what should and should not be included in such guidelines. While the results of this study can be used to consider the consistency of guidelines used across borders, it is nevertheless important for evaluators to check with and follow their local professional and organizational guidelines to ensure evaluators remain compliant with local laws and ethics codes.

While this review has uncovered a range of approaches to address best practices, there is a common understanding that the guidelines must meet the best interests of the children, regardless of the variability that continues to exist in the definition and application of the best interest principle. The quest to address the best interest of the child is laudable, regardless of the complexity of attempting to achieve this standard across the various guidelines. This review helps to shed light on the various considerations and thus provides clarity on how best interest is being considered internationally.

An important next step would be further refinement of the best interest principle by an international expert group, as this ‘open norm’ principle is challenging to interpret in actual practice. What do we consider, from the perspective of developmental psychological science, to be in the best interests of the child, in various situations? The link between the best interests, the evaluative criteria for the evaluation, and the assessment methods chosen for the evaluation can only be strengthened if we become more explicit about the best interests, and this will also serve the quality of judicial decision-making.

## Data availability statement

The raw data supporting the conclusions of this article will be made available by the authors, without undue reservation.

## Author contributions

JZ and TL conceived the project, undertook the collection and analysis of the data, wrote the manuscript, and edited the final version with SB, CR, and MS. All authors were involved in the formulation of the study questions and the interpretation of the findings. All authors contributed to the article and approved the submitted version.

## Conflict of interest

The authors declare that the research was conducted in the absence of any commercial or financial relationships that could be construed as a potential conflict of interest.

## Publisher’s note

All claims expressed in this article are solely those of the authors and do not necessarily represent those of their affiliated organizations, or those of the publisher, the editors and the reviewers. Any product that may be evaluated in this article, or claim that may be made by its manufacturer, is not guaranteed or endorsed by the publisher.

## Footnotes

^1^Due to time constraints, we were not able to receive guidelines from Australia, but had Australian expert input for the analysis of the guidelines from other countries.

^2^The authors are aware that the APA Guidelines for Child Custody Evaluation in Family Law Proceedings as well as the AFCC Model Standards for Child Custody Evaluations are currently being revised and soon to be published. As the revised versions, however, were not yet available when conducting this analysis, this work is based on the current versions of the guidelines.

^3^In the field of investigative interviewing the use of props has been shown to increase suggestion and erroneous reports of abuse (e.g., Poole et al., 2011).
