# Supplementary material for: International perspective on guidelines and policies for child custody and child maltreatment risk evaluations: A preliminary comparative analysis across selected countries in Europe and North America
Source: Front Psychol. 2022 Sep 7;13:900058. doi: 10.3389/fpsyg.2022.900058 (PMC9586013; doi:10.3389/fpsyg.2022.900058)
Supplement: Supplementary file 1 [file Table_1.DOCX]

**Guidelines for Child Custody and Child Maltreatment Risk Evaluations from an International Perspective: A Comparative Analysis**

Jelena Zumbach^1*^, Sonja Brubacher^2^, Frank Davis^3^, Corine de Ruiter^4^, Jane L. Ireland^5^, Kathleen McNamara^6^, Martta October^7^, Michael Saini^8^, Renate Volbert^1, 9^, Taina Laajasalo^11, 7^

* Correspondence:
j.zumbach@phb.de

Supplementary Material

# Supplementary Table

***Table S1.*** Country-by-country descriptions of qualifications of and training provided for evaluators in child custody and child maltreatment risk evaluations

| **Canada** |
| --- |
| ***Who conducts expert evaluations in family law and which types of cases are typically addressed?***  There are lots of variations across provinces and there are differences between child protection and parenting plan disputes (formerly called “child custody disputes” prior to the amendments of the Divorce Act in 2021) as these typically are covered by two separate ministries / legislations.  It is common in family law litigation in Canada for a mental health professional, or a team of professionals from a clinic, to be asked to provide a parenting plan assessment as well as child maltreatment investigations. There are different mental health professionals involved in conducting these assessments, including social workers, psychologists, and psychiatrists. As a generalization, assessments performed by social workers tend to be more factual, whereas assessments conducted by psychologists or psychiatrists tend to be more clinical. Parenting plan assessments are most often conducted with the consent of both parties or at the court’s request, but they may also be performed by an assessor retained by one party.  ***Which training options/requirements are provided for evaluators?***  Some assessors have taken courses on conducting forensic family law assessments and have had supervised clinical experience in this work; they may have extensive professional experience in conducting assessments for the court, and may conduct many assessments a year. Other assessments, however, may be carried out by professionals who have little or no academic background or supervised experience in conducting assessments, and who may do only a few assessments a year as a “sideline” to a therapeutically oriented practice.  ***Do legal statutes determine necessary qualifications for evaluators? If yes, which ones?***  In most Canadian jurisdictions, there is legislative provision for the court to order some type of assessment, investigation, or home study.  The content of parenting assessments, the purposes for which they may be requested and the test to obtain them are subject to rules that vary by jurisdiction and are usually set out in the provincial or territorial legislation on domestic relations.  In British Columbia, section 211(1) of the Family Law Act gives the court discretion to appoint an individual to assess one or more of the following:   1. the needs of a child in relation to a family law dispute; 2. the views of a child in relation to a family law dispute; 3. the ability and willingness of a party to a family law dispute to satisfy the needs of a child.   Section 211(2)(a) stipulates that the assessor “must be a family justice counsellor, a social worker or another person approved by the court.”  In Alberta, parenting assessments were formerly carried out under Practice Note 7, “Use of Independent Parenting Experts.” This Practice Note was repealed and replaced in 2012 by Practice Notes 7 and 8, which respectively provide procedural guidelines for “Interventions” and for “Parenting Time/Parenting Responsibilities Assessments.” Practice Note 7 provides for interventions by parenting experts, whose role is to “describe what is happening in the family and/or with the children.” Parenting experts engaged under Practice Note 7 do not provide opinions or recommendations as to the best interests of the children or as to issues such as custody and access. They provide information of limited scope to assist the court with decision-making, including voice of the child reports and psychological evaluations of the parents, and may also provide mediation, parenting coordination and therapeutic services to individuals and families.  In Ontario, assessments are governed by section 30 of the Children’s Law Reform Act, which allows the court to: (1) … appoint a person who has technical or professional skill to assess and report to the court on the needs of the child and the ability and willingness of the parties or any of them to satisfy the needs of the child. Pursuant to s-s. (5), the court may require “the parties, the child and any other person” to attend for assessment. In the event that a person fails to attend, the section provides that: (6) … the court may draw such inferences in respect of the ability and willingness of any person to satisfy the needs of the child as the court considers appropriate.  Ontario’s Courts of Justice Act addresses clinical investigations undertaken by the Office of the Children’s Lawyer (OCL). Although these investigations are initiated at the request of the court, the OCL has the discretion to decline to become involved. Section 112 of the Act states that:    (1) In a proceeding under the Divorce Act (Canada) or the Children’s Law Reform Act in which a question concerning custody of or access to a child is before the court, the Children’s Lawyer may cause an investigation to be made and may report and make recommendations to the court on all matters concerning custody of or access to the child and the child’s support and education.  ***Do further means exist to implement an evidence-based approach in the evaluation process? If yes, which ones?***  Although there is a common core of elements in most assessments, there is great diversity in the nature and quality of assessments. There is not a single accepted methodology for conducting evaluations. |
| **Finland** |
| ***Who conducts expert evaluations in family law and which types of cases are typically addressed?***  In a custody dispute, when the district court considers that it needs more information to make a decision in the best interests of the child, it will request a report ("statement of circumstances") from the social and health authorities of the child's municipality of residence. A working group or unit set up by the municipality's social services (e.g., family law unit) is responsible for carrying out the evaluation report. Professionals doing the child custody evaluations are licensed professionals (in the majority of cases social workers, occasionally psychologists). Currently, a psychologist is seldom involved in the assessment, but can be consulted. In custody cases, consulting psychologists are usually not specialized to forensic psychology, but rather developmental psychology or clinical psychology, and are guided by the professional guidelines by their respective fields (e.g., regarding assessments of parental capacity).  Since 2015, practically all child (physical or sexual) abuse allegations are a matter of criminal law and pretrial investigation is always led by the police, in addition to Child Protective Services (CPS) evaluation. A fraction of the abuse investigations (approx.1/5) are referred to Forensic Psychology/Psychiatric Units located at the University Central Hospitals, where a forensic psychologist interviews a child and a multidisciplinary team (medical, CPS, forensic services) are involved in the assessment and writing a report to the court. In these Units, all psychologists are specialized in forensic psychology. According to several publications, prior to the establishment of these specialized units, the interviews conducted within child abuse assessments were of poor quality (see e.g., Korkman et al. (2008). Interviewing techniques and follow-up questions in child sexual abuse interviews. *European Journal of Developmental Psychology, 5*, 108–128), and professionals held beliefs unsupported by empirical data (see e.g., Finnilä-Tuohimaa et al. (2005). Connections between experience, beliefs, scientific knowledge, and self-evaluated expertise among investigators of child sexual abuse in Finland. Scandinavian Journal of Psychology). Ten years later, the quality has improved considerably with the systematic utilization of hypothesis testing and the implementation of the NICHD interview protocol (article in preparation). As opposed to child maltreatment evaluations, the content and quality of Finnish custody assessments has been little studied.  ***Which training options/requirements are provided for evaluators?***  Since 2019, a two-year specialization (30 credits) in forensic psychology (for psychologists, lawyers, judges, social workers and doctors already working in the field) is organized by Åbo Akademi and other universities. This comprehensive course also covers topics related to child abuse investigations, and to a lesser extent child custody assessments.  In 2020, special training in investigating crimes against children was made a permanent part of the continuing education offering of the Finnish Police University College. Training has been available for more than 10 years. Each  course lasts two semesters (5 credits) and is held annually for 36 students, of which 28 are from police and the rest from other relevant professions (health care, social work).  There is currently no national and comprehensive training for social workers, psychologists, and/or other professionals conducting child custody assessments. The lack of possibilities to competence building and training has recently been acknowledged.  ***Do legal statutes determine necessary qualifications for evaluators? If yes, which ones?***  Regarding child abuse allegations, the Criminal Investigation Act (Chapter 4, Section 7): ‘*To the extent possible, investigation actions directed at persons under the age of 18 years shall be assigned to investigators particularly trained in this function*’. This law applies to police officers.  Based on the Act on organizing the investigation of sexual or assault offences against children, the police can ask executive assistance from a child forensic psychiatry/ forensic psychology team *(The ‘Organising Act’ 1009/2008).* According to the Act, social and health care professionals working in the unit must have specific training to the task as well as supervision. The content of the required training is not further specified.  There are no qualifications for conducting custody assessments, but you need to be a licensed professional (usually a social worker) to be employed in a unit conducting the evaluations.  ***Do further means exist to implement an evidence-based approach in the evaluation process? If yes, which ones?***  Hypothesis testing approach and the use of the NICHD – interview protocol is integrated in all national trainings and national guidelines regarding child abuse evaluations. A national guideline for child custody evaluation processes was published in 2022 (so recently it was not possible to include it in the assessment). |
| **Germany** |
| ***Who conducts expert evaluations in family law and which types of cases are typically addressed?***  Psychological evaluations in family law cases are typically conducted by independent evaluators working in private practice. Evaluators usually conduct both child custody evaluations as well as child maltreatment investigations. Some of them work in larger practices providing collegial supervision, others work in solitary practice. Some also conduct other types of forensic evaluations, such as credibility assessment in criminal (e.g., child abuse) cases. In Germany, those psychological evaluators are usually called upon by family courts in specifically difficult cases, after a Child Protective Services (CPS) evaluation has already taken place and protective or interventive measures offered to the family were deemed not sufficiently effective. CPS usually still remain involved in the cases and many times are part of the court hearings. The psychological evaluator’s task is  to additionally provide expert opinion to the judge.  ***Which training options/requirements are provided for evaluators?***  In Germany, two training options exist. The first one is the postgraduate training to become a „Licensed Forensic Psychologist“ [FachpsychologIn für Rechtspsychologie], which aims at psychologists with a university degree in psychology (Master’s diploma) who work in forensic psychology and would like to obtain certification as a specialist psychologist in legal psychology. It is offered by the Federation of German Psychologists' Associations, i.e., the Professional Association of German Psychologists and the German Psychological Society. The focus of this postgraduate program is to provide participants with a science-based professional qualification for psychological activities in the legal system. A wide range of teaching formats is used, from theoretical seminars and work in a specialist team to individual supervision in the preparation of forensic expert evaluations (total of 400 training units of 45 minutes, 240 of these are theoretical units). The training takes place while trainees conduct forensic psychological work under supervision. A total of three years of professional activity in the field is required for graduation. The program therefore extends for a minimum time period of three years. However, only few psychologists in Germany are licensed under this program per year.  The second option is to complete a Master of Science in Forensic Psychology (offered by a few universities across Germany). The master programs in Forensic Psychology exist as both, post-graduate programs for participants holding a Master’s in another area of psychology, or as post-graduate programs for participants holding a Bachelor’s degree in psychology. Students usually develop theoretical skills in one or more of four areas, including a) Forensic assessment in child custody and child abuse cases, b) Credibility assessment, c) Offender risk assessment / criminal responsibility assessment and d) Intervention in correctional and forensic psychiatric institutions. Most of the programs include four semesters of coursework and a Master’s thesis on a forensic psychological research project.  However, none of these training options are mandatory to be appointed as evaluator by the courts. It is ultimately the court’s decision whom they appoint as expert, as long as qualifications as defined below in § Section 163 of the Act on Proceedings in Family Matters and in Matters of Non-contentious Jurisdiction are met. With an ongoing debate in Germany about the quality of psychological evaluations in family law, the courts tend to emphasize / value specific training of evaluators in the field of forensic psychology more strongly, at least in urban areas with greater availability of experts.  ***Do legal statutes determine necessary qualifications for evaluators? If yes, which ones?***  This in defined in § Section 163 of the Act on Proceedings in Family Matters and in Matters of Non-contentious Jurisdiction (FamFG): In proceedings under section 151 nos. 1 to 3, the expert evaluation shall be prepared by a suitable expert who shall have at least a psychological, psychotherapeutic, child and youth psychiatric, psychiatric, medical, educational or socio-educational professional qualification. If the expert has a pedagogical or socio-educational professional qualification, the acquisition of sufficient diagnostic and analytical knowledge shall be proven by a recognized additional qualification. In proceedings concerning the person of the child, the court may order that the expert, in the context of the evaluation, additionally takes on a mediating role.  ***Do further means exist to implement an evidence-based approach in the evaluation process? If yes, which ones?***  An expert group exists that develops and publishes the German best-practice guidelines, consisting of representatives of legal, psychological and medical associations, the German Federal Bar Association and the German Federal Chamber of Psychotherapists, supported by the German Federal Ministry of Justice and Consumer Protection and supported by the XII. Civil Senate of the Federal Court of Justice. However, the guidelines developed and published by this expert group legally remain non-binding recommendations. |
| **The Netherlands** |
| ***Who conducts expert evaluations in family law and which types of cases are typically addressed?***  Evaluations in child custody cases are conducted mostly by the Child Protection Board (in Dutch: Raad voor de Kinderbescherming; RvdK). The Child Protection Board is a national organization with area teams spread across the country. The Child Protection Board is an executive organization of the Ministry of Justice and Security.  Evaluations in child custody cases are also performed by independent psychologists and psychiatrists, often at the request of the court and mediated by the Netherlands Institute of Forensic Psychiatry and Psychology (NIFP), which is also an executive organization under the Ministry of Justice and Security.  A third option is so-called ‘Forensic mediation’ (see: https://www.forensischemediation.nl/forensische-mediation). Since 2002, the term “forensic mediation” has come into use for a method of working by an expert, appointed by the judge in divorce and access cases. The method is also referred to with the term: “Expert Report with the application of Mediation” (in Dutch: Deskundigenbericht met toepassing van Mediation). The method combines investigation and mediation with the aim of, on the one hand, providing the family court with the information required to issue the judicial decision and, on the other hand, offering parties in court the opportunity to (partially) resolve their dispute themselves.  Evaluations in child maltreatment cases are conducted mostly by the Child Protection Board (in Dutch: Raad voor de Kinderbescherming; RvdK) and by Safe Home (in Dutch: Veilig Thuis), the national agency for reporting child maltreatment and domestic violence.  ***Which training options/requirements are provided for evaluators?***  Professionals who work for the Child Protection Board have to be licensed youth and family care professionals (in Dutch: ‘jeugd en gezinsprofessional’) or licensed behavioral scientists (‘gedragswetenschappers’). This means that they have to work in accordance with the professional code that applies to them. Furthermore, they keep to the Youth Care and Youth Protection Guidelines. Many different Master and Bachelor degrees in social work, creative therapy, and cultural work qualify for licensing as a youth and family care professional. The licensing body is the Stichting Kwaliteitsregister Jeugd (SKJ).  In the “National framework for forensic youth diagnostics” (“Landelijk Kader Forensische Diagnostiek Jeugd”) the requirements for forensic evaluation in civil cases are specified. Experts conducting civil forensic evaluations must meet the following requirements:  - BIG *(this is the government licensing body for health care professionals, including medical doctors, nurses, clinical psychologists, etc., CdR*) registration (and for psychiatrists: included in the specialist register as (child and youth) psychiatrist) or NVO registration as educational psychologist (in Dutch: orthopedagoog-generalist) or NIP (*NIP is the Dutch Psychological Society, CdR*) registration as psychologist for children and youth.  - not subject to any legal procedure at the time of the evaluation in relation to possible disability or unfitness for duty;  - participates in relevant continuing education and reflection.  The Netherlands Institute of Forensic Psychiatry and Psychology (NIFP) requires psychiatrists or psychologists to have completed so-called Pro Justitia training. To work as a Pro Justitia evaluator, someone must be registered with the NRGD, the Dutch Register of Judicial Experts.  Requirements for registration as a forensic mediator are:  -The Family Mediation course is a basic requirement. It must be recognized by Mediators Federation Netherlands (MfN)  -Master’s degree  -5-day training offered by the Forensic Mediation Foundation, which includes modules on legal procedure, conflict management and report writing.  ***Do legal statutes determine necessary qualifications for evaluators? If yes, which ones?***  The Youth Act (“Jeugdwet”) is the overarching legal statute that governs the entire youth welfare system, including Child Protection Board, family guardianship organizations and youth care institutions. In the Youth Act itself, there are no specific statutes that list qualifications, these are part of the Quality Frameworks / guidelines.  ***Do further means exist to implement an evidence-based approach in the evaluation process? If yes, which ones?***  “Safe Home” (in Dutch: Veilig Thuis) is the Dutch hotline to be called in case of concerns regarding a possible case of child abuse or domestic violence. It was founded on January 1st, 2015. Safe Home employs social workers, behavioral scientists, and medical doctors. Currently, there are 26 regional Safe Home organizations. These investigate allegations of child abuse and domestic violence. The work processes at Safe Home are guided by the ‘VNG- model Handelingsprotocol” (Handling Protocol). This protocol doesn’t specify how child investigative interviews should be conducted. We surveyed 158 professionals employed at the 26 Safe Home organizations and found that they do not use evidence-based interview protocols (Erens et al., 2020). On the contrary, some professionals reported using interviewing methods for which there is no empirical validation, for instance, using props such as anatomically correct dolls.  The Safe Home organizations also do not use a uniform, research-based approach for the assessment of risk of future child maltreatment. No specific training is required, except for registration with professional bodies, such as Stichting Kwaliteitsregister Jeugd. In recent years, the NICHD interview protocol has been implemented at one Safe Home organisation in South-Limburg by a research team from Maastricht University (PI: C. de Ruiter). The implementation has been evaluated by comparing 38 NICHD interviews with 30 control interviews. NICHD interviews contained more invitations and fewer suggestive questions compared to control interviews. Furthermore, children reported more details related to the reported incident when they were interviewed with the NICHD protocol compared to control interviews. The paper that reports on this study is published in *Applied Cognitive Psychology*: “The NICHD Interview Protocol Used by Dutch Child Protection Workers: Effects on Interview Style, Children’s Reported Information and Susceptibility to Suggestion” (Erens et al., 2021). |
| **United Kingdom** |
| ***Who conducts expert evaluations in family law and which types of cases are typically addressed?***  Psychologists, psychiatrists and Independent Social Workers (ISW) generally complete evaluations. For adults, they tend to focus on forensic issues of violence (e.g. domestic, general violence, sexual violence) and how safe it is for children. For child assessments, this can include concerns over their behaviour in the same way (e.g. safety towards other children/others) but more generally they are focused on the impact of the child being exposed to parental challenges and obtaining an outline of their needs. Psychiatrists will capture medical evidence, an ISW will provide an opinion independent to Local Authority social workers, with psychologists completing psychological and psychological risk assessments,  Procedure rules dictate what an expert report should include but do not define what an expert is by any regulatory body standards. This is arguably a criticism of the system. For example, there are suggestions that psychologists should be regulated via the HCPC (Health and Care Professions Council - a regulatory body in the UK for certain practitioners) or via Chartership with the BPS (British Psychological Society – for academics giving expert evidence). Medical experts are governed by medical societies, as medical doctor titles are protected (e.g. GMC – General Medical Council).  ***Which training options/requirements are provided for evaluators?***  These are locally driven and not at a national level. There is not formal mandated training required to provide expert evidence. In addition, ‘psychologist’ is not a protected term. Although we can encourage HCPC (i.e. regulator) membership but there are many instances where they are not regulated. Any training options are led by the expert, Thus, the opt in to events on giving testimony etc. but none of this is mandatory.  For example, the 2016 joint BPS and FJC (Family Justice Council) guidance notes how Courts should *expect* that all psychologists based in the UK providing evidence to be regulated by the HCPC and /or have (or are eligible for) Chartered status with the BPS, or meet the criteria as set down in the FJC standards document for overseas experts. A key focus is on having some degree of regulation.  ***Do legal statutes determine necessary qualifications for evaluators? If yes, which ones?***  No. For example, they willrequire a psychologist for psychological assessments but do not dictate qualifications. The CV is judged by those instructing.  ***Do further means exist to implement an evidence-based approach in the evaluation process? If yes, which ones?***  No. |
| **United States** |
| ***Who conducts expert evaluations in family law and which types of cases are typically addressed?***  Suspicions of child maltreatment are evaluated by Departments of Social Services, and their case workers are paid by the state. Child custody evaluations may be conducted by private practitioners, agency workers, or court services evaluators.  This varies by state.  Fees are usually paid by the parties, but some states provide state pay, or partial state pay for custody evaluations when the parties are below a certain income level.  Child custody evaluators often assess claims of poor parenting that do not rise to the level of a suspicion of child abuse/neglect, or sometimes Social Services has already investigated the matter and dismissed the case as unsubstantiated or unfounded.  Suspicions of child maltreatment may remain a serious concern within the family, and evaluators often called upon to evaluate these issues. In Colorado, child custody evaluators and investigators must have training in child maltreatment and domestic violence both initially and on an ongoing basis. It varies widely by state what the requirements, if any, are. For example, the state of Ohio is about to enact state-provided child custody training which will include training on child maltreatment. **The answers below refer only to child custody evaluations.**  ***Which training options/requirements are provided for evaluators?***  Training options/requirements vary by state. There are a few forensic psychology graduate programs around the country, but not many. Most obtain training through continuing education programs (specific workshops) offered by associations such as AFCC and APA, or from universities, private organizations (such as the Steve Frankel Group), and state/local groups. There are very few internship opportunities. Most new evaluators must find their own supervision. Only a few states have supervision requirements. Some states provide initial and continuing training for evaluators, but many do not.  For example, in California, you must be a licensed mental health professional, and have 40 hours of coursework training (including interpersonal violence training) across a range of various topics/domains. Additionally, you must be 'supervised' by a qualified child custody evaluator on 4 child custody evaluations prior to being able to work independently as a private child custody evaluator.  Keep in mind that there are custody evaluators who are Court employees and work for the public sector as well.  ***Do legal statutes determine necessary qualifications for evaluators? If yes, which ones?***  Generally, yes, but those statutes vary by state. Most professionals doing child custody evaluations are licensed mental health professionals (psychologists, social workers, counselors), but that is not a requirement in every state.  In California, the necessary qualifications for custody evaluators have been established and governed by the California Rules of Court.  In Colorado there is a two-tier system. Only licensed mental health professionals may conduct comprehensive evaluations referred to as a "Parental Responsibilities Evaluation (PRE)."  These evaluators must complete 40 hours of continuing education every two years, which is not provided by the state. The second tier is referred to as a "Child and Family Investigators (CFI)."  CFIs conduct brief, cost-limited investigations and offer opinions and recommendations about custody. The amount CFIs can charge for the investigation is capped by the state. CFIs are not required to be a licensed mental health professional, and many are not. They may be attorneys, probation officers, retired teachers, etc. They must complete an initial 40-hour training program that is provided by the state. Both PREs and CFIs must also complete training in domestic violence and child maltreatment, which is not provided by the state. In Colorado, a child custody evaluator (parental responsibilities evaluator) must meet the following educational training requirements according to statute.  (a) The effects of divorce and remarriage on children, adults, and families;  (b) Appropriate parenting techniques;  (c) Child development, including cognitive, personality, emotional, and psychological development;  (d) Child and adult psychopathology;  (e) Applicable clinical assessment techniques; and  (f) Applicable legal and ethical requirements of parental responsibilities evaluation.  ***Do further means exist to implement an evidence-based approach in the evaluation process? If yes, which ones?***  This area is a work in progress. As noted previously, the Association of Family Conciliation Courts puts on trainings year around to help evaluators implement and maintain evidence-based approaches to their forensic work. More research on the efficacy of methods used in evaluations and the kinds of services that are frequently recommended by evaluators would be helpful. |
